# Consensus-based Recommendations on the Management of Immunosuppression After Squamous Cell Carcinoma Diagnosis in Kidney Transplant Recipients: An International Delphi Consensus Statement

**DOI:** 10.1097/TXD.0000000000001893

**Published:** 2025-12-17

**Authors:** Melodi Javid Whitley, Jake X. Wang, Jeremy Chapman, Aiko P.J. de Vries, Sindhu Chandran, Colleen M. Glennon, Hector M. Madariaga, Robert P. Carroll, John Booth, Beatrice Concepcion, Lionel Couzi, Sacha A. De Serres, Alden Doyle, Suzanne Forbes, Jonathan M. Gleadle, Sian Griffin, Lakshman Gunaratnam, Gaurav Gupta, Julie Ho, Peter Hughes, Nicole Isbel, Dev K. Jegatheesan, Gareth Jones, Dirk Kuypers, Brian Lee, Wai Lim, Phil Mason, Maria Meneghini, Itunu Owoyemi, Swati Rao, Alan Salama, Adnan Sharif, Olivier Thaunat, Raj Thuraisingham, Kate Wyburn, Julie M. Yabu, Anokhi Jambusaria-Pahlajani, Matthew J. Bottomley, Sarah T. Arron

**Affiliations:** 1 Department of Dermatology, Duke University School of Medicine, Durham, NC.; 2 Department of Dermatology, University Hospitals Cleveland Medical Center, Case Western Reserve University, Cleveland, OH.; 3 Consultant Emeritus, Renal Unit, Westmead Hospital, Westmead, NSW, Australia.; 4 Leiden Transplant Center, Division of Nephrology, Department of Medicine, Leiden University Medical Center and Leiden University, Leiden, the Netherlands.; 5 Department of Medicine, Division of Nephrology, Comprehensive Transplant Center, Cedars-Sinai Medical Center, Los Angeles, CA.; 6 Department of Medicine, Tufts University School of Medicine, Burlington, MA.; 7 Transplantation Immunogenetics Service, Lifeblood, Australian Redcross Blood Service, Sydney, NSW, Australia.; 8 Barts Health NHS Trust, Royal London Hospital, London, United Kingdom.; 9 Section of Nephrology, Department of Medicine, The University of Chicago, Chicago, IL.; 10 Department of Nephrology, Transplantation, Dialysis and Apheresis, Bordeaux University Hospital, Bordeaux, France.; 11 Renal Division, Department of Medicine, Laval University, Quebec, QC, Canada.; 12 Division of Nephrology, University of Virginia Health System, Charlottesvilla, VA.; 13 Department of Nephrology and Transplantation, The Royal London Hospital, Bart’s Health NHS Trust, London, United Kingdom.; 14 Department of Renal Medicine, Southern Adelaide Local Health Network, Flinders Medical Centre, Bedford Park, SA, Australia.; 15 University Hospital of Wales, Cardiff, United Kingdom.; 16 Matthew Mailing Centre for Translational Transplant Studies, Multi-Organ Transplant Program, London Health Sciences Centre, London, ON, Canada.; 17 Division of Nephrology, Virginia Commonwealth University, Richmond, VA.; 18 Department of Medicine, University of Manitoba, Winnipeg, MB, Canada.; 19 Department of Nephrology, The Royal Melbourne Hospital, Parkville, VIC, Australia.; 20 Department of Kidney and Transplant Services, Princess Alexandra Hospital, Brisbane, QLD, Australia.; 21 Department of Kidney and Transplant Services, Princess Alexandra Hospital, Brisbane, QLD, Australia.; 22 University College London Department of Renal Medicine, Royal Free London NHS Foundation Trust, London, United Kingdom.; 23 Department of Nephrology and Renal Transplantation, University Hospitals Leuven, University of Leuven, Leuven, Belgium.; 24 Division of Nephrology, Department of Medicine, University of Texas at Austin, Austin, TX.; 25 Medical School, University of Western Australia, Perth, WA, Australia.; 26 Oxford Transplant Centre, The Churchill Hospital, Oxford, United Kingdom.; 27 Nephrology and Kidney Transplantation Laboratory, Vall d'Hebron Research Institute (VHIR), Barcelona, Spain; Department of Nephrology and Kidney Transplantation, University Hospital Vall d'Hebron, Barcelona, Spain.; 28 Department of Kidney Medicine, Cleveland Clinic, Cleveland, OH.; 29 Division of Nephrology, University of Virginia Health System, Charlottesvilla, VA.; 30 University College of London Centre for Kidney and Bladder Health, Division of Medicine, Faculty of Medical Sciences, University College London, London, UK.; 31 Department of Nephrology, University Hospitals Birmingham, Birmingham, UK.; 32 Service de Transplantation, Néphrolgie et Immunologie Clinique, Hôpital Edouard Herriot, Hospices Civils de Lyon, Lyon, France.; 33 Department of Renal Medicine and Transplantation, Barts Health NHS Trust, London, UK.; 34 Department of Renal Medicine, Royal Prince Alfred Hospital, Camperdown, NSW, Australia.; 35 Division of Nephrology, Department of Medicine, University of California, Los Angeles, CA.; 36 Division of Dermatology, Dell Medical School, University of Texas at Austin, Austin, TX.; 37 Chinese Academy of Medical Sciences (CAMS) Oxford Institute, Nuffield Department of Medicine, University of Oxford, Oxford, United Kingdom.; 38 Oxford Kidney and Transplant Unit, Churchill Hospital, Oxford University Hospitals NHS Foundation Trust, Oxford, United Kingdom.; 39 Peninsula Dermatology Medical Group, Burlingame, CA.

## Abstract

**Background.:**

Posttransplant immunosuppression in kidney transplant recipients is associated with an increased risk of developing cutaneous squamous cell carcinoma (CSCC), contributing to significant morbidity and mortality. Various dermatological and immunosuppression modulation strategies have been identified that may reduce the risk of CSCC, both in primary and secondary prevention settings. Recent recommendations have provided consensus regarding dermatological approaches to prevent CSCC. Comparable transplant nephrology recommendations to guide immunosuppression modulation for CSCC prevention are currently lacking, leading to marked variation in practice.

**Methods.:**

To address this knowledge gap, 46 international transplant nephrology experts participated in a 3-round Delphi survey to develop consensus recommendations for CSCC secondary prevention based on the actinic damage and skin cancer index stages of CSCC.

**Results.:**

The panel of experts reached consensus to consider a change in immunosuppression after multiple low-risk invasive CSCC (stage 5a, 1/y >3 y) and encouraged collaboration with dermatology to optimize dermatologic preventative care after the first CSCC. There was also consensus to prioritize azathioprine modification where this is present in an immunosuppressive regimen.

**Conclusions.:**

This study provides the first international consensus recommendations for management of immunosuppression in kidney transplant recipients at discrete stages of CSCC. Additional prospective studies are necessary to determine the optimal management of immunosuppression in this patient population. These recommendations have been endorsed by the Board of the American Society of Transplantation.

## INTRODUCTION

Cutaneous squamous cell carcinoma (CSCC) is the most common skin cancer in solid organ transplant recipients (SOTRs), ultimately affecting up to half of patients by 20 y posttransplant.^1^ CSCC behaves more aggressively in SOTRs compared with immunocompetent populations even when adjusted by clinicopathologic stage.^2,3^ After the first CSCC, kidney transplant recipients (KTRs) have an increased risk of developing subsequent lesions, with the median time from first to second tumor estimated at 12.7 mo.^4^ Advanced tumor stage and the presence of multiple CSCC are both risk factors for metastatic spread, which is associated with poor survival.^3,5^ The increase in incidence, tumor burden, and metastasis are attributable to immunosuppressive medications that have off-target effects on tumor immunosurveillance and direct mutagenic properties.^6^ With increasing global incidence of kidney transplantation, particularly among older populations, morbidity and mortality from posttransplant CSCC are expected to grow, in line with that seen in nonimmunosuppressed populations.^7-10^

Multiple studies suggest that timely changes in immunosuppressive regimen, along with dermatologic interventions such as increased screening and chemoprevention, may impact subsequent skin cancer behavior.^11-14^ It is therefore critical to develop strategies to prevent first CSCC (primary prevention) and subsequent tumors (secondary prevention) in KTRs. With regards to immunosuppression modification, previous studies support that CSCC incidence can be lowered by reduction or cessation of immunosuppressive agents with direct carcinogenic effects such as azathioprine, but no consensus guidelines exist for management of immunosuppression for secondary prevention of CSCC, resulting in significant variation in prevention strategies.^11,15-18^ A recent multi-institution European study found that both the frequency and type of immunosuppression reduction (eg, whether calcineurin inhibitor [CNI] or antimetabolite was reduced) differed significantly within and across transplant centers following CSCC in KTRs.^19^ This highlights the current equipoise in decision-making. Thus, physicians would benefit from clear guidance on clinical best practice for immunosuppression modification in this setting.

In the absence of high quality randomized clinical trials, expert consensus can provide valuable guidance. A recent expert consensus statement on dermatologic recommendations for prevention of CSCC in SOTRs established a novel actinic damage and skin cancer index (AD-SCI) system categorizing patients from early skin photodamage (stage 1) to first invasive low-risk CSCC (stage 4) to high-risk CSCC (stage 6; Table 1).^20^ The expert panel of transplant dermatologists reached consensus on recommendations for AD-SCI stages 1–3 and 5–6, but no consensus could be reached for stage 4: at the first low-risk CSCC. This was potentially because of the unclear transition between stages 1 and 3, which can be managed within the dermatology practice, and stages 5–6, for which multidisciplinary care and systemic interventions were recommended.

**TABLE 1.** Actinic damage and skin cancer index (AD-SCI)

Stage	Description
1	Photodamaged skin only
2	Discrete actinic keratoses
3	Field cancerization
4	First low-risk^*a*^ SCC
5	Multiple low-risk^*a*^ CSCC
5a	Slow rate^*b*^
5b	Fast rate^*b*^
6	High risk^*a*^ CSCC

A categorical staging system for common clinical scenarios in patients with CSCC. Stages 1–3 includes photodamaged skin only to precancerous field cancerization, whereas stages 4–6 begins after the first low-risk invasive CSCC. Adapted from Massey et al.^20^

^*a*^Low-risk CSCC is defined as AJCC8 T1–T2 and BWH tumor classification T1–T2a. High risk is defined as AJCC8 T3 or BWH T2b with ~20% risk of nodal metastasis.

^*b*^Slow rate of tumor accrual is defined as ~1 per 12 mo over 36 mo and fast rate of tumor accrual is defined as ~5 per 12 mo.

AJCC8, American Joint Committee on Cancer, 8th Edition; BWH, Brigham and Women’s Hospital; CSCC, cutaneous squamous cell carcinoma; SCC, squamous cell carcinoma.

The present study was designed to build on these recommendations by integrating the international transplant nephrology expert perspective. The goal was to establish international Delphi consensus recommendations for the secondary prevention of CSCC in KTRs, focusing on stages after first low-risk CSCC. This article reports these expert consensus recommendations, which have been endorsed by the Board of the American Society of Transplantation.

## METHODS

The ITSCC-DAN (Immunosuppression Transplant Skin Cancer Collaborative Dermatology and Nephrology) Working Group convened at the 2022 ITSCC Symposium. The working group reviewed the transplant dermatologist Delphi consensus study with the goal of integrating transplant nephrologists into the Delphi consensus recommendations for secondary prevention of CSCC in KTRs.^20^ This survey study was approved by the Duke University Institutional Review Board Pro00112333.

The survey was conducted between July 2023 and August 2024. The initial round was open for 2 mo as participants were recruited during the peer esteem snowball technique process, while subsequent rounds occurred over a 1-mo response period.^21^

### Steering Committee

The steering committee was composed of 4 transplant dermatologists (M.J.W., J.X.W., A.J.P., S.T.A.) and 4 transplant nephrologists (S.C., J.C., A.D.V., M.J.B.). S.T.A. and M.J.B. served as co-chairs of the steering committee. The steering committee determined the scope and methods of the study, designed the Delphi surveys, reviewed and interpreted the data from each Delphi round, and formulated the conclusions of the study.

### Expert Panelists

Expert panelists were identified using peer esteem snowball technique.^21^ Transplant nephrologists on the steering committee and additional advisors (R.P.C., H.M.M.) served as seed nominators. Experts were defined as English-speaking nephrologists with at least 5 y of posttraining practice experience and ongoing responsibility for the long-term management (>1 y posttransplant) of KTRs. All qualified respondents were asked to nominate up to 5 additional experts for inclusion in the study. Panelists were invited to participate via e-mail in a Research Electronic Data Capture screening survey. E-mail invitations to the Research Electronic Data Capture surveys were sent up to 4 times to each prospective respondent at each round of the study. Panelists were given the option to provide their names; however, these were stored separately from the survey responses, which were aggregated anonymously. Panelists who completed all 3 rounds of the survey were given the option to participate as coauthors on this article.

### Survey Design

First round data collection requested information about respondent demographics, including relevant clinical and academic experience, and practice setting/location. The first round of the Delphi Survey asked whether respondents would take action in regard to skin cancer prevention for KTRs with history of at least 1 invasive squamous cell carcinoma corresponding to AD-SCI stages 4, 5a, 5b, and 6 (Table 1). If the respondent indicated that a change in immunosuppression should be made, they were queried as to which immunosuppressive agent(s) would be changed and how.

The second survey round focused on dermatology engagement to optimize preventative care. Respondents were also asked more specific questions regarding immunosuppression changes for KTRs with AD-SCI stage 4–6 disease for specific regimens.

In the third and final round, panelists were asked which factors would significantly motivate or deter them from making specific immunosuppression changes recommended in round 2. Reasons for disagreement were queried. At all survey rounds, respondents were provided with multiple choice questions as well as opportunities to propose new items and add to their responses in an open-text format. Panelists were made aware of the dermatology consensus recommendations and presented with the AD-SCI convention but were not given other literature.^19^ They were also informed of the results after each round.

All 3 survey round questions are included in the **Supplemental Materials (SDC**, https://links.lww.com/TXD/A814). M.J.B. piloted the Delphi survey rounds and these responses were not used in the calculation of the final consensus. S.C., J.C., and A.D.V. participated in the Delphi survey rounds and their responses were used in the calculation of the final consensus.

### Definition of Consensus and Statistical Analysis

We predetermined a consensus threshold of 80% agreement and near consensus threshold of 70% agreement, corresponding to previous standards.^20^ Negative consensus was defined as <20% agreement. Descriptive statistics were performed after each round for multiple choice questions; thematic analysis was undertaken on free responses to identify common threads.

The steering committee reviewed the data after each round and a summary was provided to all respondents before the subsequent round. When items met consensus, more detailed questions regarding next best steps were developed. When consensus was not met, clinical reasoning or alternative approaches were queried further.

The article was written in accordance with the recently published ACcurate COnsensus Reporting Document guidelines on the reporting of consensus methods in biomedicine (**Checklist**, **SDC**, https://links.lww.com/TXD/A814).^22,23^

## RESULTS

### Respondent Recruitment and Characteristics

There were 6 initial seeds in the peer esteem snowballing approach (Table 2). These initial seeds nominated 44 potential respondents in the second stage, followed by 34, 24, and 11 nominees in the 3rd, 4th, and final stages, respectively. In total, 119 invitations were sent out to potential panelists. Of these, 53 nominated experts (45% of those invited) completed the eligibility survey and 48 (91%) of these were determined to be eligible. Forty-six panelists completed the first round of the survey. Of these, 65% reported working in an academic setting, 11% reported being hospital-based, and 24% reported working in both academic and hospital-based settings (Table 3). There was diverse geographic representation with 37% of respondents from the United States, 20% from the United Kingdom, 15% from the European Union, 17% from Australia, and 11% from Canada. All but 1 respondent reported affiliation with a transplant center. Thirty percent of respondents reported 21+ y in practice with 24%, 26%, and 20% reporting 15–20, 10–15, and 5–10 y in practice, respectively. Over 95% of respondents reported membership in a transplant professional society. Respondents reported a median of 45 (range, 1–460) publications relating to kidney transplantation and half of respondents reported at least 1 publication relating to posttransplant skin cancer. Thirty-one panelists completed all 3 rounds of the Delphi, with no significant shift in demographics between rounds.

**TABLE 2.** Recruitment and retention of Delphi panelists

First stage				Second stage				Third stage				Fourth stage				Final stage				Total		
Initial seeds	Survey rounds complete				Survey rounds complete				Survey rounds complete				Survey rounds complete				Survey rounds complete			Survey rounds complete		
	1	1 + 2	1 + 2 + 3	Nominations	1	1 + 2	1 + 2 + 3	Nominations	1	1 + 2	1 + 2 + 3	Nominations	1	1 + 2	1 + 2 + 3	Nominations	1	1 + 2	1 + 2 + 3	1	1 + 2	1 + 2 + 3
6	4	3	3	44	20	16	13	34	13	11	10	24	7	5	4	11	2	1	1	46	36	31
	66.67%	50.00%	50.00%		45.45%	36.36%	29.55%		38.24%	32.35%	29.41%		29.17%	20.83%	16.67%		18.18%	9.09%	9.09%			

Recruitment and retention of Delphi panelists. Panelists were identified via the peer esteem snowball technique. Experts were defined as English-speaking nephrologists with at least 5 y of posttraining practice experience and ongoing responsibility for the long-term (>1 y posttransplant) immunosuppression management of kidney transplant patients. Six initial seeds nominated 44 potential respondents in the second stage, followed by 34, 24, and 11 nominees in the 3rd, 4th, and final stages, respectively. In total, 119 invitations were sent out to potential panelists. Forty-six respondents completed the first round, followed by 36 and 31 in subsequent rounds.

**TABLE 3.** Expert panelist characteristics

46 total panelists	
Practice setting, n (%)	
Academic/university	30 (65)
Hospital/multispecialty	5 (11)
Both	11 (24)
Region, n (%)	
United States	17 (37)
Canada	5 (11)
United Kingdom	9 (20)
European Union	7 (15)
Australia	8 (17)
Professional society membership, n (%)	
American Society of Transplantation	30 (65)
American Society of Transplant Surgeons	1 (2)
The Transplantation Society	15 (32)
Other	23 (50)
None	2 (4)
Years in practice, n (%)	
5–10	9 (20)
10–15	12 (26)
15–20	11 (24)
>20	14 (30)
Transplant center affiliation (n = yes)	45 (98)
Kidney transplant publications	
Minimum	1
Maximum	460
Median	45
Mode	20
Skin cancer in kidney transplant publications	
Minimum	0
Maximum	60
Median	0.5
Mode	0

(A) Panelists represented both academic/university and hospital/multispecialty-based practice settings. (B) Panelists represented transplant nephrologists from the United States, Canada, United Kingdom, European Union, and Australia. (C) Panelist experience based on years in practice. (D) Panelist experience based on publication record.

### Consensus Recommendations

**FIGURE 1.**
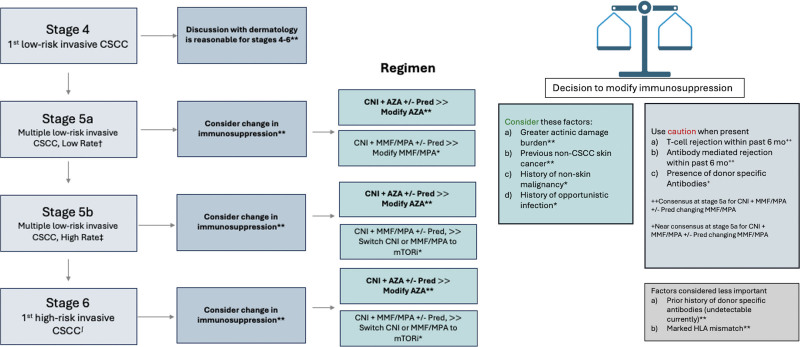
Summary recommendations for kidney transplant recipients (KTRs) with CSCC. This figure shows a decision pathway for transplant nephrologists caring for KTRs with CSCC, using actinic damage and skin cancer index (AD-SCI) stages to risk-stratify patients. Factors favoring a change in immunosuppression and possible reasons to use caution are also indicated. **Consensus, *near consensus, †1 CSCC per year >3 y, ‡5 CSCC >12 mo, ∫American Joint Committee on Cancer, 8th Edition. Stage T3/Brigham and Women’s Hospital T2b. AZA, azathioprine; CNI, calcineurin inhibitor; CSCC, cutaneous squamous cell carcinoma; MMF/MPA, mycophenolate mofetil/mycophenolic acid; mTORi, mammalian target of rapamycin inhibitor; Pred, prednisone.

The Delphi consensus recommendations are summarized in Figure 1.

### Liaison With Dermatology

There was consensus after any invasive CSCC (stages 4–6) that an appropriate next step for the prevention of additional CSCC would be to discuss the patient with his or her dermatologist and encourage them to optimize dermatologic preventative care (eg, field therapy, oral nicotinamide, oral acitretin).

### Immunosuppression Modification

**FIGURE 2.**
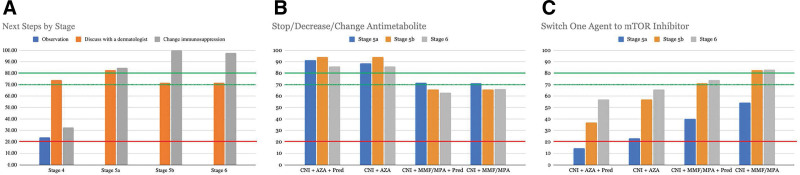
Consensus recommendations for changes to immunosuppression. A, Delphi recommendations for next steps by stage. B, Delphi recommendations for changes to antimetabolite by stage and immunosuppression regimen. C, Delphi recommendations for a switch to mTOR inhibitor by stage and immunosuppression regimen. Consensus set at 80% agreement (solid green), near consensus at 70% (dotted green), and negative consensus at 20% (solid red). AZA, azathioprine; CNI, calcineurin inhibitor; MMF/MPA, mycophenolate mofetil/mycophenolic acid; mTOR, mammalian target of rapamycin; Pred, prednisone.

The panel did not recommend revision of immunosuppression at stage 4 (first low-risk CSCC), but they achieved consensus on a change in immunosuppression at stages 5a–b and 6 (multiple low-risk CSCC or 1 high-risk CSCC; Figure 2A). Specifically, at stages 5–6, the panel recommended that patients on an immunosuppression regimen including azathioprine should have the azathioprine reduced, stopped, or changed to an alternative antimetabolite (ie, mycophenolate mofetil/mycophenolic acid [MMF/MPA]; Figure 2B). For patients at stages 5b and 6 on MMF/MPA and a CNI, there was at least near consensus to change 1 agent to a mammalian target of rapamycin inhibitor (mTORi; Figure 2C).

Finally, the panel explored factors that may influence the decision to change immunosuppression. There was consensus at all stages that prior (not current) evidence of donor-specific antibodies and degree of HLA mismatch would not influence a decision to change immunosuppression in the setting of CSCC (Figure 1). The panel recommended that recent history of T-cell–mediated rejection (consensus), antibody-mediated rejection (consensus), and/or current evidence of donor-specific antibodies (near consensus) would represent relative contraindications to changing the antimetabolite for patients at stage 5a on a MMF/MPA-based regimen. Conversely, there was consensus that overall, actinic keratosis burden and a history of non-CSCC skin cancer would favor a change in immunosuppression, with near consensus that history of nonskin malignancy and prior opportunistic infection should also be considered as factors favoring immunosuppression change.

## DISCUSSION

Despite long-standing recognition of the significant contribution of CSCC to posttransplant morbidity and mortality among KTR, and the key role of immunosuppression itself in this process, there are no specific guidelines or uniform consensus regarding management of immunosuppressive regimens to reduce future tumor burden. Indeed, there is a dearth of adequately powered interventional trials to address this knowledge gap and guide practice, leading to clinical equipoise (COAST [Contemporary Outcomes After Skin cancer in Transplant] multicenter cohort study, 2025, preprint).^19^ For this reason, this study aimed to develop consensus recommendations regarding the secondary prevention of CSCC from a global panel of transplant nephrologists experienced in the long-term management of KTR and posttransplant CSCC.

The Delphi process reached consensus on several management recommendations in AD-SCI stages 4–6. Notably, transplant nephrologists reached consensus that immunosuppression modification is favored for stages 5a and higher but there was no consensus to make such change at stage 4. While a recent Delphi study of transplant dermatologists failed to reach consensus on recommendations for Stage 4 disease, transplant nephrologists in this study agreed that for patients at stage 4, discussion with their dermatologist is appropriate.^20^ This not only highlights the importance of early multidisciplinary care for SOTRs who develop CSCC but also indicates the cross-disciplinary therapeutic equipoise that exists at this stage. Although high quality studies are limited in number, some trials argue for intervening at earlier stages, that is, after the first low-risk invasive CSCC or after slow rate of development, at least for certain regimens. For example, 2 independent trials suggested the secondary prevention benefit of conversion from a CNI to sirolimus was greater in patients with a single CSCC than after multiple CSCC.^12,13^ Additional prospective trials are needed to refine management.

The panel felt that interdisciplinary collaboration between transplant nephrologists and dermatologists was a critical part of patient care, with consensus reached that discussion with dermatology is an appropriate next step after the development of any invasive CSCC. In the previous Delphi panel of dermatologists, there was consensus to initiate discussions with transplant physicians at stages 5–6 in their Delphi, which aligns with our study’s findings that nephrologists consider changing immunosuppression at stage 5.^20^

The panel recommended antimetabolites as the first medications to be reduced or stopped when modifying immunosuppression. For patients on a dual or triple drug regimen including azathioprine and a CNI, consensus was reached to reduce, discontinue or switch azathioprine at stages 5a–6. For patients on mycophenolate regimens, there was near consensus at stage 5a to change the mycophenolate. Azathioprine is well-known for its association with UV-A photosensitivity, which has been shown to reverse after switching to MMF/MPA, and induces a unique mutational signature, likely explaining why it was considered the first-choice agent to change.^18,24^ Another possibility is that azathioprine is an indicator of stable, long-term immunosuppression, which nephrologists are more comfortable adjusting. While azathioprine is used less commonly in modern immunosuppressive regimens, having been superseded by MPA-based preparations, it remains in common use among long-term KTR, in whom the risk of CSCC is greatest. Still, although observational studies and meta-analyses have associated azathioprine use with enhanced CSCC risk, the evidence is not definitive for the benefit of its cessation in secondary prevention of CSCC.^25^

It is also notable that physicians advocated a change to an mTORi only after multiple low-risk CSCC, or single high-risk CSCC, despite interventional data suggesting this approach is most efficacious after a single lesion.^12,13^ This likely represents physician reticence to commence these agents in view of their limited tolerability, and risk of rebound effect with cessation.^25^ Furthermore, although the available randomized controlled trials focus on switching the CNI to mTORi, our panelists elected to give the clinician the option of switching either the CNI or antimetabolite to mTORi at higher stages (near consensus).^12,13^ The rationale was a concern that switching CNI to mTORi increases the risk of de novo donor-specific antibody formation and potential rejection^26^; so, for patients who have proteinuria or poor allograft function, modification of the antimetabolite was sometimes preferred.

Additionally, newer immunosuppressants such as belatacept may be reasonable alternatives to CNIs. In a single-center, retrospective study, switching to belatacept from a CNI reduced incidence of CSCC in KTRs; however, a study of patients on belatacept in the de novo setting did not show this protective effect.^27,28^ Given the limited available evidence and potential barriers to accessing belatacept in some regions, it was not included in this international Delphi.

Our study is subject to the limitations of the Delphi process. The Delphi panel represents a relatively small sample size; however, the number of panelists is comparable to previously published Delphi consensus statements in this area.^19^ Although response rates were high, not all panelists finished all 3 rounds. Reassuringly, there was no evidence of a selection bias among those who completed the study, in terms of a shift in demography. Our expert transplant nephrologists predominantly practice in developed countries with a Caucasian majority; therefore, this may introduce bias into our recommendations, which may differ in regions with darker skin phototypes predominate and in more resource-scarce settings. Lastly, although round 3 of the Delphi attempted to determine the most important factors that would influence the decision to change immunosuppression such as overall actinic burden and history of nonskin malignancy, there may be additional factors not considered. Patient preference was beyond the scope of this study, but as the final component of the transplant dermatology shared care “triad,” future work will be needed to explore patient perspectives and priorities on this topic. Patient preference is an important consideration and immunosuppression modification should only take place as part of a shared decision-making process.^25^ Some patients may not tolerate even a small increased risk of graft loss; however, a thorough discussion balancing the morbidity and mortality of CSCC (20% risk of nodal metastasis at stage 6) is important to help patients decide. Finally, while the general themes of these recommendations may be relevant to other transplant populations, this Delphi consensus statement is specific for KTRs. For example, while a switch from azathioprine to mycophenolate and early collaboration with dermatology is likely to be favored in thoracic transplant recipients as well, the ideal timing for immunosuppression modification may differ in these patients.

These consensus recommendations are not a statement of standard of care, particularly considering international practice variations, which may be impacted by various factors, including insurance coverage. Rather, the results represent frameworks to be used by clinicians to tailor treatment for each individual patient.

In summary, this study provides the first consensus recommendations for management of immunosuppression for secondary prevention of CSCC. However, the paucity of interventional data, alongside the predicted increase in posttransplant CSCC burden, highlights the urgent need for prospective studies in this area to optimize KTR quality and quantity of life, particularly in the setting of early-stage (stage 4) disease where interventions may be of greatest benefit.

## ACKNOWLEDGMENTS

The authors thank the panelists for their insights and responses. Also, the authors thank Ranita Silver for her assistance with survey distribution.

## Supplementary Material


