# Supplementary material for: Consensus-based Recommendations on the Management of Immunosuppression After Squamous Cell Carcinoma Diagnosis in Kidney Transplant Recipients: An International Delphi Consensus Statement
Source: Transplant Direct. 2025 Dec 17;12(1):e1893. doi: 10.1097/TXD.0000000000001893 (PMC12711373; doi:10.1097/TXD.0000000000001893)
Supplement: Supplementary file 1 [file txd-12-e1893-s001.pdf]

Supplemental Table 1. The International Immunosuppression Transplant Skin Cancer Collaborative Dermatology & Nephrology (ITSCC-DAN) Management of Immunosuppression after Squamous cell carcinoma in renal Transplant (MIST) Study Panelists

Name	Affiliation 1	Affiliation 2	COIs
Alden Doyle, MD	Division of Nephrology, University of Virginia Health System, Charlottesvilla, VA, USA		None
Adnan Sharif MD, MBChB, FRCP	University Hospital Birmingham, University of Birmingham, UK		None
Alan Salama MBBS PhD FRCP	UCL Centre for Kidney and Bladder Health, Royal Free Hospital, London, UK		None
Beatrice Concepcion, MD	Section of Nephrology, Department of Medicine, The University of Chicago, IL, USA		None
Brian Lee MD	Division of Nephrology, Department of Medicine, University of Texas at Austin, TX, USA		None
Dev Krish Jegatheesan MBBS, FRACP	Department of Kidney and Transplant Services, Princess Alexandra Hospital, Brisbane, Australia	Faculty of Medicine, University of Queensland, Brisbane, Australia	None
Dirk Kuypers, MD PhD	Department of Nephrology and Renal Transplantation, University Hospitals Leuven, University of Leuven, Belgium		None
Hector M Madariaga MD	Department of Medicine, Tufts University School of Medicine, Burlington, MA, USA		None
Gareth Jones, PhD	University College London Department of Renal Medicine, Royal Free London NHS Foundation Trust, London, United Kingdom		
Gaurav Gupta MD	Division of Nephrology, Virginia Commonwealth University, Richmond, VA, USA		Advisory Board: CareDx, Eurofins-Viracor, Mallinckrodt, Natera, Novartis; Research support: Merck, Veloxis, NIH/NIAID, NIH/NIDDK; Honoraria: Alexion, CareDx, Natera
Julie Ho MD, FRCPC	Internal Medicine, University of Manitoba, Winnipeg, Manitoba, Canada		Funding: Canadian Institutes of Health Research
John Booth MBBS, PhD	Barts Health NHS Trust, Royal London Hospital, London, UK		None
Jonathan Mark Gleadle BM BCh, MA, DPhil, FRCP, FRACP	Department of Renal Medicine, Southern Adelaide Local Health Network, Flinders Medical Centre, Bedford Park, SA, Australia	Flinders University, College of Medicine and Public Health, Flinders Health and Medical Research Institute, Adelaide, SA, Australia	None
Julie M. Yabu MD, MTM	Division of Nephrology, Department of Medicine, University of California, Los Angeles, CA, USA		None
Kate Wyburn MBBS, FRACP, Phd	Department of Renal Medicine, Royal Prince Alfred Hospital, Camperdown, Sydney, Australia		None

Lakshman Gunaratnm, MD, MSc, FRCPC	Matthew Mailing Centre for Translational Transplant Studies, Multi-Organ Transplant Program, London Health Sciences Centre, London, ON, Canada	Division of Nephrology, Department of Medicine, Schulich School of Medicine and Dentistry, Western University, London, ON, Canada	Advisory Board: Paladin Pharma Inc.; Board of Directors: Canadian Society of Transplantation; Honoraria: Paladin Pharma Inc.
Lionel Couzi, MD, PhD	Department of Nephrology, Transplantation, Dialysis and Apheresis, Bordeaux University Hospital, Bordeaux 33000, France	CNRS-UMR 5164 ImmunoConcEpT, Bordeaux University, Bordeaux 33076, France	Funding: Astellas, Chiesi, Sandoz, Sanofi; Honoraria: Astellas, Chiesi, Hansa, MDS, Novartis, Ostuka, Sandoz; Consultancy Fees: BMS, Chiesi, Hansa, Novartis, Takeda
Maria Meneghini, MD, PhD	Vall 'Hebron University Hospital , Barcelona, Spain		None
Nicole Isbel MBBS, PhD	Department of Kidney and Transplant Services, Princess Alexandra Hospital, Brisbane, Australia	Faculty of Health, Medicine, and Behavioural Science, University of Queensland, Brisbane, Australia	None
Olivier Thaunat MD, PhD	Service de Transplantation, Néphrolgie et Immunologie Clinique, Hôpital Edouard Herriot, Hospices Civils de Lyon, 5 Place d'Arsonval, Lyon FRANCE	Université Claude Bernard Lyon I, UFR de médecine Lyon Est, Campus Rockfeller, Lyon, FRANCE	None
Itunu Owoyemi MBBS	Department of Kidney Medicine, Cleveland Clinic, Cleveland, Ohio, USA		Advisory Board: National Kidney Foundation Transplant Advisory Board; Funding: VeloSano Pilot Grant Award, Robert A Winn Excellent in Clinical Trials: Career Development Award
Peter Hughes MBBS (Hons), FRACP, PhD	Department of Nephrology, The Royal Melbourne Hospital, Parkville, Victoria, Australia	Department of Medicine, The University of Melbourne, Melbourne, Victoria, Australia	None
Phil Mason PhD MBBS FRCP	Oxford Transplant Centre, Oxford, UK		None
Raj Thuraisingham FRC	Barts Health NHS Trust, London, UK		None
Sacha A. De Serres MD, SM, FRCPC	Renal Division, Department of Medicine, Laval University, Quebec, QC, Canada		None
Sian Griffin MB BChir, PhD, FRCP	University Hospital of Wales, Cardiff, UK		None
Swati Rao MD	Division of Nephrology, University of Virginia Health System, Charlottesvilla, VA, USA		None
Suzanne Forbes MBChB	Department of Nephrology and Transplantation, The Royal London Hospital, Bart's Health NHS Trust, London, UK		None
Wai Lim MBBS, FRACP, PhD	Medical School, University of Western Australia, Perth, Australia	Department of Renal Medicine, Sir Charles Gairdner Hospital, Perth, Australia	None

Eligibility

Thank you for agreeing to participate in this survey.

Please answer the following questions to determine your eligibility for the Delphi panel to achieve consensus among nephrologists on management of cutaneous squamous cell carcinoma in renal transplant recipients.

Are you a practicing Nephrologist?	◯ Yes<br>◯ No
Do you have ongoing responsibility for the long-term immunosuppression management of kidney transplant patients?<br>Long-term is defined as after 1 year post-transplant	◯ Yes<br>◯ No
Have you practiced for at least 5 years since completion of postgraduate medical training in nephrology?	◯ Yes<br>◯ No
Are you comfortable completing this survey in English?	◯ Yes<br>◯ No

Thank you for completing this part of the survey. Based on your experience, you are eligible for the study.

The purpose of this study is to achieve consensus among nephrologists on management of cutaneous squamous cell carcinoma in renal transplant recipients. This is an IRB-approved (Duke University, USA) survey-based study using a Delphi method to achieve consensus. You have been recommended as a nephrologist with expertise in the care of renal transplant recipients. This is the first round of the survey and will be followed by 2-3 additional rounds based on initial responses. We anticipate that each round will take no more than 15 minutes to complete.

Thank you for your participation. Please press submit to continue.

Thank you for completing the eligibility questions for this study. We appreciate your time and interest! Unfortunately, the inclusion criteria were not met. To be included in future outreach or emails regarding skin cancer after organ transplantation, please enter your email address:	__________________________

Delphi Survey: Round 1

Demographic Information

Please provide the following demographic information.

How many years have you been in practice since completion of postgraduate medical training in nephrology?

- ◯ 5-10 years
- ◯ 10-15 years
- ◯ 15-20 years
- ◯ more than 20 years

Are you a member of any of the following societies? Check all that apply:

- ☐ American Society of Transplantation
- ☐ American Society of Transplant Surgeons
- ☐ The Transplantation Society
- ☐ International Society for Heart and Lung Transplantation
- ☐ Other
- ☐ None

Please specify other societies:

____

Approximately how many publications do you have on the topic of kidney transplantation?

____

Approximately how many publications do you have specifically on the topic of skin cancer after transplantation?

____

What is your primary practice setting? Check all that apply:

- ☐ academic/university affiliated practice
- ☐ hospital/multispecialty group
- ☐ private practice
- ☐ government
- ☐ other

Please specify other practice setting:

____

Are you affiliated with a transplant center?

- ◯ Yes
- ◯ No

What transplant center are you affiliated with?

____

What country do you practice in?

- ◯ Afghanistan
- ◯ Albania
- ◯ Algeria
- ◯ American Samoa
- ◯ Andorra
- ◯ Angola
- ◯ Anguilla
- ◯ Antarctica
- ◯ Antigua and Barbuda
- ◯ Argentina
- ◯ Armenia
- ◯ Aruba
- ◯ Australia
- ◯ Austria
- ◯ Azerbaijan
- ◯ The
- ◯ Bahrain
- ◯ Bangladesh
- ◯ Barbados
- ◯ Belarus
- ◯ Belgium
- ◯ Belize
- ◯ Benin
- ◯ Bermuda
- ◯ Bhutan
- ◯ Bolivia
- ◯ Bosnia and Herzegovina
- ◯ Botswana
- ◯ Bouvet Island
- ◯ Brazil
- ◯ British Indian Ocean Territory
- ◯ British Virgin Islands
- ◯ Brunei
- ◯ Bulgaria
- ◯ Burkina Faso
- ◯ Burma
- ◯ Burundi
- ◯ Cambodia
- ◯ Cameroon
- ◯ Canada
- ◯ Cape Verde
- ◯ Cayman Islands
- ◯ Central African Republic
- ◯ Chad
- ◯ Chile
- ◯ China
- ◯ Christmas Island
- ◯ Cocos (Keeling) Islands
- ◯ Colombia
- ◯ Comoros
- ◯ Democratic Republic of the Congo
- ◯ Cook Islands
- ◯ Costa Rica
- ◯ Cote d'Ivoire
- ◯ Croatia
- ◯ Cuba
- ◯ Curacao
- ◯ Cyprus
- ◯ Czech Republic
- ◯ Denmark
- ◯ Djibouti
- ◯ Dominica
- ◯ Dominican Republic
- ◯ Ecuador
- ◯ Egypt
- ◯ El Salvador
- ◯ Equatorial Guinea
- ◯ Eritrea
- ◯ Estonia

◯ Ethiopia
◯ Falkland Islands (Islas Malvinas)
◯ Faroe Islands
◯ Fiji
◯ Finland
◯ France
◯ French Guiana
◯ French Polynesia
◯ French Southern and Antarctic Lands
◯ Gabon
◯ Gambia
◯ Gaza Strip
◯ Georgia
◯ Germany
◯ Ghana
◯ Gibraltar
◯ Greece
◯ Greenland
◯ Grenada
◯ Guadeloupe
◯ Guam
◯ Guatemala
◯ Guernsey
◯ Guinea
◯ Guinea-Bissau
◯ Guyana
◯ Haiti
◯ Heard Island and McDonald Islands
◯ Holy See (Vatican City)
◯ Honduras
◯ Hong Kong (SAR China)
◯ Hungary
◯ Iceland
◯ India
◯ Indonesia
◯ Iran
◯ Iraq
◯ Ireland
◯ Isle of Man
◯ Israel
◯ Italy
◯ Jamaica
◯ Japan
◯ Jersey
◯ Jordan
◯ Kazakhstan
◯ Kenya
◯ Kiribati
◯ South Korea
◯ Kosovo
◯ Kuwait
◯ Kyrgyzstan
◯ Laos
◯ Latvia
◯ Lebanon
◯ Lesotho
◯ Liberia
◯ Libya
◯ Liechtenstein
◯ Lithuania
◯ Luxembourg
◯ Macau (SAR China)
◯ Macedonia
◯ Madagascar
◯ Malawi
◯ Malaysia
◯ Maldives
◯ Mali
◯ Malta
◯ Marshall Islands
◯ Martinique

◯ Mauritania
◯ Mauritius
◯ Mayotte
◯ Mexico
◯ Federated States of Micronesia
◯ Moldova
◯ Monaco
◯ Mongolia
◯ Montenegro
◯ Montserrat
◯ Morocco
◯ Mozambique
◯ Namibia
◯ Nauru
◯ Nepal
◯ Netherlands
◯ New Caledonia
◯ New Zealand
◯ Nicaragua
◯ Niger
◯ Nigeria
◯ Niue
◯ Norfolk Island
◯ Northern Mariana Islands
◯ Norway
◯ Oman
◯ Pakistan
◯ Palau
◯ Panama
◯ Papua New Guinea
◯ Paraguay
◯ Peru
◯ Philippines
◯ Pitcairn Islands
◯ Poland
◯ Portugal
◯ Puerto Rico
◯ Qatar
◯ Reunion
◯ Romania
◯ Russia
◯ Rwanda
◯ Saint Barthelemy
◯ Saint Helena, Ascension, and Tristan da Cunha
◯ Saint Kitts and Nevis
◯ Saint Lucia
◯ Saint Martin
◯ Saint Pierre and Miquelon
◯ Saint Vincent and the Grenadines
◯ Samoa
◯ San Marino
◯ Sao Tome and Principe
◯ Saudi Arabia
◯ Senegal
◯ Serbia
◯ Seychelles
◯ Sierra Leone
◯ Singapore
◯ Sint Maarten
◯ Slovakia
◯ Slovenia
◯ Solomon Islands
◯ Somalia
◯ South Africa
◯ South Georgia and the Islands
◯ South Sudan
◯ Spain
◯ Sri Lanka
◯ Sudan
◯ Suriname
◯ Svalbard

◯ Swaziland
◯ Sweden
◯ Switzerland
◯ Syria
◯ Taiwan
◯ Tajikistan
◯ Tanzania
◯ Thailand
◯ Timor-Leste
◯ Togo
◯ Tokelau
◯ Tonga
◯ Trinidad and Tobago
◯ Tunisia
◯ Turkey
◯ Turkmenistan
◯ Turks and Caicos Islands
◯ Tuvalu
◯ Uganda
◯ Ukraine
◯ United Arab Emirates
◯ United Kingdom
◯ United States
◯ United States Minor Outlying Islands
◯ Uruguay
◯ Uzbekistan
◯ Vanuatu
◯ Venezuela
◯ Vietnam
◯ Virgin Islands
◯ Wallis and Futuna
◯ West Bank
◯ Western Sahara
◯ Yemen
◯ Zambia
◯ Zimbabwe

What city do you practice in?

____

Contact Information
Please note: Your responses will be kept confidential and will not be attributable to any specific panelist when results are published and/or presented. Contact information is used to send panelists summary results from each Delphi Survey and to request participation in the subsequent round. Additionally, this will allow us to appropriately credit your participation as an expert panelist in any resulting publications and/or presentations.

First Name: ______________________

Last Name: ______________________

E-Mail Address: ______________________

Do you wish to be a named contributor in any resulting publications and/or presentations?
- ◯ Yes
- ◯ No

The following questions address medical management for cutaneous squamous cell carcinomas (cSCC) in renal transplant recipients (RTRs). Please select options you would consider the most acceptable in medical management. For the following questions, please assume the specific tumor has already been treated surgically with negative margins.

Please consider a typical renal transplant recipient (stable graft function on a stable, standard immunosuppressive regimen in your center, with no history or current evidence of humoral sensitization) you see in your clinic.

Definition of Actinic Damage - Skin Cancer Index (AD-SCI) Stages:

Stage 4 First invasive low-risk* cSCC (AJCC8 T1-T2; BWH T1-T2a)
Stage 5 Multiple invasive low-risk* cSCC (AJCC8 T1-T2; BWH T1-T2a)
5a Slow rate @ approximately 1 per 12 months over last 36 months
5b Fast rate @ approximately 5 over last 12 months
Stage 6 High-risk cSCC (AJCC8 T3; BWH T2b) *~20% risk of nodal metastasis
Massey PR et al. Consensus-Based Recommendations on the Prevention of Squamous Cell Carcinoma in Solid Organ Transplant Recipients: A Delphi Consensus Statement. JAMA Dermatol. 2021 Oct 1;157(10):1219-1226. doi: 10.1001/jamadermatol.2021.3180. PMID: 34468690; PMCID: PMC9937447.

A renal transplant recipient has been diagnosed with their first ever invasive low-risk cSCC (Stage 4). Assume the specific tumor has already been treated appropriately. Which of the following do you consider to be an appropriate next step for the prevention of additional cSCC? Choose all that apply.
- ☐ Observation only
- ☐ Discuss patient with dermatologist and encourage them to optimize dermatologic preventative care (e.g. field therapy, oral nicotinamide, oral acitretin)
- ☐ Consider a change in immunosuppressive agent(s)

Which immunosuppressive medication(s) would you stop, start, or revise? [check all that apply]
If you are considering multiple possible regimen changes you will have the opportunity to clarify your approach in an area provided for "additional comments" at the end of this section.
- ☐ Prednisone/Prednisolone
- ☐ Azathioprine
- ☐ Mycophenolate
- ☐ Tacrolimus
- ☐ Cyclosporine
- ☐ mTor Inhibitor
- ☐ Other

Please specify "Other" medication(s): ______________________

How would you modify the prednisone/prednisolone?	◯ Stop<br>◯ Start<br>◯ Decrease dose/trough<br>◯ Increase dose/trough
How would you modify the azathioprine?	◯ Stop<br>◯ Start<br>◯ Decrease dose/trough<br>◯ Increase dose/trough
How would you modify the mycophenolate?	◯ Stop<br>◯ Start<br>◯ Decrease dose/trough<br>◯ Increase dose/trough
How would you modify the tacrolimus?	◯ Stop<br>◯ Start<br>◯ Decrease dose/trough<br>◯ Increase dose/trough
How would you modify the cyclosporine?	◯ Stop<br>◯ Start<br>◯ Decrease dose/trough<br>◯ Increase dose/trough
How would you modify the mTOR inhibitor?	◯ Stop<br>◯ Start<br>◯ Decrease dose/trough<br>◯ Increase dose/trough
Which of the following patient/allograft factors do you consider in planning revision of immunosuppression?	☐ Time post transplant<br>☐ Prior use of depleting induction therapy<br>☐ Other immunosuppressive medications<br>☐ Other non-immunosuppressive medications (photosensitizers, acitretin, nicotinamide etc)<br>☐ Immunologic risk/donor matching<br>☐ Graft function<br>☐ Prior history of rejection<br>☐ Prior history of sun-exposure/degree of sun-damage<br>☐ Patient Preference<br>☐ Other
Please specify other factors:	__________________________________
Please provide any additional comments regarding revision of immunosuppression agent(s) in this scenario:	__________________________________

The following questions address medical management for cutaneous squamous cell carcinomas (cSCC) in renal transplant recipients (RTRs). Please select options you would consider the most acceptable in medical management. For the following questions, please assume the specific tumor has already been treated surgically with negative margins.

Please consider a typical renal transplant recipient (stable graft function on a stable, standard immunosuppressive regimen in your center, with no history or current evidence of humoral sensitization) you see in your clinic.

Definition of Actinic Damage - Skin Cancer Index (AD-SCI) Stages:

Stage 4 First invasive low-risk* cSCC (AJCC8 T1-T2; BWH T1-T2a)
Stage 5 Multiple invasive low-risk* cSCC (AJCC8 T1-T2; BWH T1-T2a)
5a Slow rate @ approximately 1 per 12 months over last 36 months
5b Fast rate @ approximately 5 over last 12 months
Stage 6 High-risk cSCC (AJCC8 T3; BWH T2b) *~20% risk of nodal metastasis
Massey PR et al. Consensus-Based Recommendations on the Prevention of Squamous Cell Carcinoma in Solid Organ Transplant Recipients: A Delphi Consensus Statement. JAMA Dermatol. 2021 Oct 1;157(10):1219-1226. doi: 10.1001/jamadermatol.2021.3180. PMID: 34468690; PMCID: PMC9937447.

A renal transplant recipient has been diagnosed with mulitple invasive low-risk cSCC at a slow rate (Stage 5a). Assume the specific tumors have already been treated appropriately. Which of the following do you consider to be an appropriate next step for the prevention of additional cSCC? Choose all that apply.

☐ Observation only
☐ Discuss patient with dermatologist and encourage them to optimize dermatologic preventative care (e.g. field therapy, oral nicotinamide, oral acitretin)
☐ Consider a change in immunosuppressive agent(s)

Which immunosuppressive medication(s) would you stop, start, or revise? [check all that apply]
If you are considering multiple possible regimen changes you will have the opportunity to clarify your approach in an area provided for "additional comments" at the end of this section.

☐ Prednisone/Prednisolone
☐ Azathioprine
☐ Mycophenolate
☐ Tacrolimus
☐ Cyclosporine
☐ mTor Inhibitor
☐ Other

Please specify other medication(s):

____

How would you modify the prednisone/prednisolone?

○ Stop
○ Start
○ Decrease dose/trough
○ Increase dose/trough

How would you modify the azathioprine?

○ Stop
○ Start
○ Decrease dose/trough
○ Increase dose/trough

How would you modify the mycophenolate?

○ Stop
○ Start
○ Decrease dose/trough
○ Increase dose/trough

How would you modify the tacrolimus?

○ Stop
○ Start
○ Decrease dose/trough
○ Increase dose/trough

How would you modify the cyclosporine?

◯ Stop
◯ Start
◯ Decrease dose/trough
◯ Increase dose/trough

How would you modify the mTOR inhibitor?

◯ Stop
◯ Start
◯ Decrease dose/trough
◯ Increase dose/trough

Which of the following patient/allograft factors do you consider in planning revision of immunosuppression?

☐ Time post transplant
☐ Prior use of depleting induction therapy
☐ Other immunosuppressive medications
☐ Other non-immunosuppressive medications (photosensitizers, acitretin, nicotinamide etc)
☐ Immunologic risk/donor matching
☐ Graft function
☐ Prior history of rejection
☐ Prior history of sun-exposure/degree of sun-damage
☐ Patient Preference
☐ Other

Please specify other factors:

____

Please provide any additional comments regarding revision of immunosuppression agent(s) in this scenario:

____

The following questions address medical management for cutaneous squamous cell carcinomas (cSCC) in renal transplant recipients (RTRs). Please select options you would consider the most acceptable in medical management. For the following questions, please assume the specific tumor has already been treated surgically with negative margins.

Please consider a typical renal transplant recipient (stable graft function on a stable, standard immunosuppressive regimen in your center, with no history or current evidence of humoral sensitization) you see in your clinic.

Definition of Actinic Damage - Skin Cancer Index (AD-SCI) Stages:

Stage 4 First invasive low-risk* cSCC (AJCC8 T1-T2; BWH T1-T2a)
Stage 5 Multiple invasive low-risk* cSCC (AJCC8 T1-T2; BWH T1-T2a)
5a Slow rate @ approximately 1 per 12 months over last 36 months
5b Fast rate @ approximately 5 over last 12 months
Stage 6 High-risk cSCC (AJCC8 T3; BWH T2b) *~20% risk of nodal metastasis
Massey PR et al. Consensus-Based Recommendations on the Prevention of Squamous Cell Carcinoma in Solid Organ Transplant Recipients: A Delphi Consensus Statement. JAMA Dermatol. 2021 Oct 1;157(10):1219-1226. doi: 10.1001/jamadermatol.2021.3180. PMID: 34468690; PMCID: PMC9937447.

A renal transplant recipient has been diagnosed with multiple invasive low-risk cSCC at a fast rate (Stage 5b). Assume the specific tumors have already been treated appropriately. Which of the following do you consider to be an appropriate next step for the prevention of additional cSCC? Choose all that apply.

☐ Observation only
☐ Discuss patient with dermatologist and encourage them to optimize dermatologic preventative care (e.g. field therapy, oral nicotinamide, oral acitretin)
☐ Consider a change in immunosuppressive agent(s)

Which immunosuppressive medication(s) would you stop, start, or revise? [check all that apply]<br>If you are considering multiple possible regimen changes you will have the opportunity to clarify your approach in an area provided for "additional comments" at the end of this section.	☐ Prednisone/Prednisolone<br>☐ Azathioprine<br>☐ Mycophenolate<br>☐ Tacrolimus<br>☐ Cyclosporine<br>☐ mTor Inhibitor<br>☐ Other
Please specify other medication(s):	__________________________________
How would you modify the prednisone/prednisolone?	◯ Stop<br>◯ Start<br>◯ Decrease dose/trough<br>◯ Increase dose/trough
How would you modify the azathioprine?	◯ Stop<br>◯ Start<br>◯ Decrease dose/trough<br>◯ Increase dose/trough
How would you modify the mycophenolate?	◯ Stop<br>◯ Start<br>◯ Decrease dose/trough<br>◯ Increase dose/trough
How would you modify the tacrolimus?	◯ Stop<br>◯ Start<br>◯ Decrease dose/trough<br>◯ Increase dose/trough
How would you modify the cyclosporine?	◯ Stop<br>◯ Start<br>◯ Decrease dose/trough<br>◯ Increase dose/trough
How would you modify the mTOR inhibitor?	◯ Stop<br>◯ Start<br>◯ Decrease dose/trough<br>◯ Increase dose/trough
Which of the following patient/allograft factors do you consider in planning revision of immunosuppression?	☐ Time post transplant<br>☐ Prior use of depleting induction therapy<br>☐ Other immunosuppressive medications<br>☐ Other non-immunosuppressive medications (photosensitizers, acitretin, nicotinamide etc)<br>☐ Immunologic risk/donor matching<br>☐ Graft function<br>☐ Prior history of rejection<br>☐ Prior history of sun-exposure/degree of sun-damage<br>☐ Patient Preference<br>☐ Other
Please specify other factors:	__________________________________

Please provide any additional comments regarding revision of immunosuppression agent(s) in this scenario: __________________________________

The following questions address medical management for cutaneous squamous cell carcinomas (cSCC) in renal transplant recipients (RTRs). Please select options you would consider the most acceptable in medical management. For the following questions, please assume the specific tumor has already been treated surgically with negative margins.

Please consider a typical renal transplant recipient (stable graft function on a stable, standard immunosuppressive regimen in your center, with no history or current evidence of humoral sensitization) you see in your clinic.

Definition of Actinic Damage - Skin Cancer Index (AD-SCI) Stages:

Stage 4 First invasive low-risk* cSCC (AJCC8 T1-T2; BWH T1-T2a)
Stage 5 Multiple invasive low-risk* cSCC (AJCC8 T1-T2; BWH T1-T2a)
5a Slow rate @ approximately 1 per 12 months over last 36 months
5b Fast rate @ approximately 5 over last 12 months
Stage 6 High-risk cSCC (AJCC8 T3; BWH T2b) *~20% risk of nodal metastasis
Massey PR et al. Consensus-Based Recommendations on the Prevention of Squamous Cell Carcinoma in Solid Organ Transplant Recipients: A Delphi Consensus Statement. JAMA Dermatol. 2021 Oct 1;157(10):1219-1226. doi: 10.1001/jamadermatol.2021.3180. PMID: 34468690; PMCID: PMC9937447.

A renal transplant recipient has been diagnosed with their first ever invasive high-risk cSCC (Stage 6). Assume the specific tumor has already been treated appropriately. Which of the following do you consider to be an appropriate next step for the prevention of additional cSCC? Choose all that apply.

- ☐ Observation only
- ☐ Discuss patient with dermatologist and encourage them to optimize dermatologic preventative care (e.g. field therapy, oral nicotinamide, oral acitretin)
- ☐ Consider a change in immunosuppressive agent(s)

Which immunosuppressive medication(s) would you stop, start, or revise? [check all that apply]
If you are considering multiple possible regimen changes you will have the opportunity to clarify your approach in an area provided for "additional comments" at the end of this section.

- ☐ Prednisone/Prednisolone
- ☐ Azathioprine
- ☐ Mycophenolate
- ☐ Tacrolimus
- ☐ Cyclosporine
- ☐ mTor Inhibitor
- ☐ Other

Please specify other medication(s): __________________________

How would you modify the prednisone/prednisolone?

- ○ Stop
- ○ Start
- ○ Decrease dose/trough
- ○ Increase dose/trough

How would you modify the azathioprine?

- ○ Stop
- ○ Start
- ○ Decrease dose/trough
- ○ Increase dose/trough

How would you modify the mycophenolate?

- ○ Stop
- ○ Start
- ○ Decrease dose/trough
- ○ Increase dose/trough

How would you modify the tacrolimus?

- ○ Stop
- ○ Start
- ○ Decrease dose/trough
- ○ Increase dose/trough

How would you modify the cyclosporine?	◯ Stop<br>◯ Start<br>◯ Decrease dose/trough<br>◯ Increase dose/trough
How would you modify the mTOR inhibitor?	◯ Stop<br>◯ Start<br>◯ Decrease dose/trough<br>◯ Increase dose/trough
Which of the following patient/allograft factors do you consider in planning revision of immunosuppression?	☐ Time post transplant<br>☐ Prior use of depleting induction therapy<br>☐ Other immunosuppressive medications<br>☐ Other non-immunosuppressive medications (photosensitizers, acitretin, nicotinamide etc)<br>☐ Immunologic risk/donor matching<br>☐ Graft function<br>☐ Prior history of rejection<br>☐ Prior history of sun-exposure/degree of sun-damage<br>☐ Patient Preference<br>☐ Other
Please specify other factors:	________________________
Please provide any additional comments regarding revision of immunosuppression agent(s) in this scenario:	________________________
If a change in immunosuppression is not considered in the preceding scenarios, at what point would you consider a change in immunosuppression?	________________________

Thank you for completing this survey, we appreciate your participation in the study! In the coming months, we will provide you with a summary of the results of the first round of the study as well as an invitation to participate in additional rounds.

We also need your help to identify additional qualified experts for participation in this study. Please recommend five additional experts for participation in this study by providing their names and email addresses below.

Expert Recommendation #1:	
First Name:	____________________
Last name	____________________
E-mail Address:	____________________
Expert Recommendation #2:	
First Name:	____________________

Last name

E-mail Address:

Expert Recommendation #3:

First Name:

Last name

E-mail Address:

Expert Recommendation #4:

First Name:

Last name

E-mail Address:

Expert Recommendation #5:

First Name:

Last name

E-mail Address:

Delphi Round 2

Please complete the survey below.

Thank you!

Thank you for your continued participation in this study.

Round 2 of the Delphi consensus panel aims to achieve consenus in areas where we neared consensus on Round 1 (Please see the summary emailed to you for details). This survey can be taken on a mobile device, but will appear clearer and may take less time on a computer.

The following questions address medical management for cutaneous squamous cell carcinomas (cSCC) in renal transplant recipients (RTRs). Please select options you consider to be the most acceptable in medical management. For the following questions, please consider a typical RTR in your clinic and assume the specific tumor has already been treated surgically with negative margins.

Definition of Actinic Damage - Skin Cancer Index (AD-SCI) Stages:

Stage 4 First invasive low-risk cSCC (AJCC8 T1-T2; BWH T1-T2a)
Stage 5 Multiple invasive low-risk cSCC (AJCC8 T1-T2; BWH T1-T2a)
5a Slow rate @ approximately 1 per 12 months over last 36 months
5b Fast rate @ approximately 5 over last 12 months
Stage 6 High-risk cSCC (AJCC8 T3; BWH T2b) ~20% risk of nodal metastasis
Massey PR et al. Consensus-Based Recommendations on the Prevention of Squamous Cell Carcinoma in Solid Organ Transplant Recipients: A Delphi Consensus Statement. JAMA Dermatol. 2021 Oct 1;157(10):1219-1226. doi: 10.1001/jamadermatol.2021.3180. PMID: 34468690; PMCID: PMC9937447.

In the first round of this survey, respondents reached consensus regarding the following statement for patients with stage 5a disease only:

An appropriate next step for the prevention of additional cSCC would be to discuss the patient with their dermatologist and encourage them to optimize dermatologic preventative care (ie field therapy, oral nicotinamide, oral acitretin).

In the following questions, please consider whether discussion with a dermatologist is also appropriate at other stages.

A renal transplant recipient has been diagnosed with their first ever invasive low-risk cSCC (Stage 4 disease).

Consider the following statement:
An appropriate next step for the prevention of additional cSCC would be to discuss the patient with their dermatologist and encourage them to optimize dermatologic preventative care (ie field therapy, oral nicotinamide, oral acitretin).

○ Agree
○ Disagree

Please indicate why you disagree with the above statement. Choose all that apply.	☐ I assume the dermatologist is already optimizing treatment<br>☐ We have an established protocol for the co-management of these patients<br>☐ It is logistically challenging to contact dermatology<br>☐ I ask the patient to talk to their dermatologist<br>☐ I am not alway notified when my patient develops their 1st cSCC<br>☐ I do not think this stage of cSCC warrants communication with dermatology<br>☐ I do not think dermatologic intervention is likely to impact cSCC risk at this stage<br>☐ Other
Please explain:	__________________________________
A renal transplant recipient has been diagnosed with stage 5b disease (multiple invasive low-risk cSCCs at a fast rate).<br><br>Consider the following statement:<br>An appropriate next step for the prevention of additional cSCC would be to discuss the patient with their dermatologist and encourage them to optimize dermatologic preventative care (ie field therapy, oral nicotinamide, oral acitretin).	◯ Agree<br>◯ Disagree
Please indicate why you disagree with the above statement. Choose all that apply.	☐ I assume the dermatologist is already optimizing treatment<br>☐ We have an established protocol for the co-management of these patients<br>☐ It is logistically challenging to contact dermatology<br>☐ I ask the patient to talk to their dermatologist<br>☐ I am not alway notified when my patient develops their 1st cSCC<br>☐ I do not think this stage of cSCC warrants communication with dermatology<br>☐ I do not think dermatologic intervention is likely to impact cSCC risk at this stage<br>☐ Other
Please explain:	__________________________________
A renal transplant recipient has been diagnosed with stage 6 disease (one or more high-risk cSCCs with ~20% risk of nodal metastasis).<br><br>Consider the following statement:<br>An appropriate next step for the prevention of additional cSCC would be to discuss the patient with their dermatologist and encourage them to optimize dermatologic preventative care (ie field therapy, oral nicotinamide, oral acitretin).	◯ Agree<br>◯ Disagree

Please indicate why you disagree with the above statement. Choose all that apply.

☐ I assume the dermatologist is already optimizing treatment
☐ We have an established protocol for the co-management of these patients
☐ It is logistically challenging to contact dermatology
☐ I ask the patient to talk to their dermatologist
☐ I am not alway notified when my patient develops their 1st cSCC
☐ I do not think this stage of cSCC warrants communication with dermatology
☐ I do not think dermatologic intervention is likely to impact cSCC risk at this stage
☐ Other

Please explain: ______________________________

The following questions address medical management for cutaneous squamous cell carcinomas (cSCC) in renal transplant recipients (RTRs). Please select options you would consider the most acceptable in medical management. For the following questions, please consider a typical RTR in your clinic and assume the specific tumor has already been treated surgically with negative margins.

Definition of Actinic Damage - Skin Cancer Index (AD-SCI) Stages:

Stage 4 First invasive low-risk cSCC (AJCC8 T1-T2; BWH T1-T2a)
Stage 5 Multiple invasive low-risk cSCC (AJCC8 T1-T2; BWH T1-T2a)
5a Slow rate @ approximately 1 per 12 months over last 36 months
5b Fast rate @ approximately 5 over last 12 months
Stage 6 High-risk cSCC (AJCC8 T3; BWH T2b) ~20% risk of nodal metastasis
Massey PR et al. Consensus-Based Recommendations on the Prevention of Squamous Cell Carcinoma in Solid Organ Transplant Recipients: A Delphi Consensus Statement. JAMA Dermatol. 2021 Oct 1;157(10):1219-1226. doi: 10.1001/jamadermatol.2021.3180. PMID: 34468690; PMCID: PMC9937447.

You are taking care of a renal transplant patient with stage 5a disease (multiple invasive low risk cSCC at a slow rate) on the immunosuppression regimen listed on each row. Please mark the next best step regarding change in immunosuppression.

	Stop/Decrease Antimetabolite	Decrease Antimetabolite and Start/Increase Prednisone	Decrease Antimetabolite and Increase Calcineurin Inhibitor	Switch to different antimetabolite	Stop Calcineurin Inhibitor and Start mTOR Inhibitor	Stop Antimetabolite and Start mTOR Inhibitor	No change
Calcineurin Inhibitor + Azathioprine + Prednisone	○	○	○	○	○	○	○
Calcineurin Inhibitor + Azathioprine	○	○	○	○	○	○	○
Calcineurin Inhibitor + MMF/MPA + Prednisone	○	○	○	○	○	○	○
Calcineurin Inhibitor + MMF/MPA	○	○	○	○	○	○	○

For a patient with stage 5a disease on a calcineurin inhibitor, azathioprine, and prednisone you chose NOT to make any changes in immunosuppression.

Consider the following statement:
I would agree with a change in immunosuppression if (choose all that apply):

- ☐ The patient is amenable to modification of immunosuppression
- ☐ There is no evidence of DSA
- ☐ There is a low level of HLA mismatch
- ☐ There has been enough time since transplant
- ☐ I have no concerns about graft function
- ☐ Patient does not have a history of rejection with this graft
- ☐ If CNI trough level is sufficiently high (top 50% of target range for your center)
- ☐ Other

Please define low HLA mismatch: ______________________

Please explain what other factors your consider: ______________________

For a patient with stage 5a disease on a calcineurin inhibitor and azathioprine you chose NOT to make any changes in immunosuppression.

Consider the following statement:
I would agree with a change in immunosuppression if (choose all that apply):

- ☐ The patient is amenable to modification of immunosuppression
- ☐ There is no evidence of DSA
- ☐ There is a low level of HLA mismatch
- ☐ There has been enough time since transplant
- ☐ I have no concerns about graft function
- ☐ Patient does not have a history of rejection with this graft
- ☐ If CNI trough level is sufficiently high (top 50% of target range for your center)
- ☐ Other

Please define low HLA mismatch: ______________________

Please explain what other factors your consider: ______________________

For a patient with stage 5a disease on a calcineurin inhibitor, MMF/MPA, and prednisone you chose NOT to make any changes in immunosuppression.

Consider the following statement:
I would agree with a change in immunosuppression if (choose all that apply):

- ☐ The patient is amenable to modification of immunosuppression
- ☐ There is no evidence of DSA
- ☐ There is a low level of HLA mismatch
- ☐ There has been enough time since transplant
- ☐ I have no concerns about graft function
- ☐ Patient does not have a history of rejection with this graft
- ☐ If CNI trough level is sufficiently high (top 50% of target range for your center)
- ☐ Other

Please define low HLA mismatch: ______________________

Please explain what other factors your consider: ______________________

For a patient with stage 5a disease on a calcineurin inhibitor and MMF/MPA you chose NOT to make any changes in immunosuppression.

Consider the following statement:
I would agree with a change in immunosuppression if (choose all that apply):

- ☐ The patient is amenable to modification of immunosuppression
- ☐ There is no evidence of DSA
- ☐ There is a low level of HLA mismatch
- ☐ There has been enough time since transplant
- ☐ I have no concerns about graft function
- ☐ Patient does not have a history of rejection with this graft
- ☐ If CNI trough level is sufficiently high (top 50% of target range for your center)
- ☐ Other

Please define low HLA mismatch:

____

Please explain what other factors your consider:

____

You are taking care of a renal transplant patient with stage 5b disease (multiple invasive low risk cSCC at a fast rate) on the immunosuppression regimen listed on each row. Please mark the next best step regarding change in immunosuppression.

	Stop/Decrease Antimetabolite	Decrease Antimetabolite and Start/Increase Prednisone	Decrease Antimetabolite and Increase Calcineurin Inhibitor	Switch to different antimetabolite	Stop Calcineurin Inhibitor and Start mTOR Inhibitor	Stop Antimetabolite and Start mTOR Inhibitor	No change
Calcineurin Inhibitor + Azathioprine + Prednisone	○	○	○	○	○	○	○
Calcineurin Inhibitor + Azathioprine	○	○	○	○	○	○	○
Calcineurin Inhibitor + MMF/MPA + Prednisone	○	○	○	○	○	○	○
Calcineurin Inhibitor + MMF/MPA	○	○	○	○	○	○	○

For a patient with stage 5b disease on a calcineurin inhibitor, azathioprine, and prednisone you chose NOT to make any changes in immunosuppression.

Consider the following statement:
I would agree with a change in immunosuppression if (choose all that apply):

- ☐ The patient is amenable to modification of immunosuppression
- ☐ There is no evidence of DSA
- ☐ There is a low level of HLA mismatch
- ☐ There has been enough time since transplant
- ☐ I have no concerns about graft function
- ☐ Patient does not have a history of rejection with this graft
- ☐ If CNI trough level is sufficiently high (top 50% of target range for your center)
- ☐ Other

Please define low HLA mismatch:

____

Please explain what other factors your consider: __________________________________

For a patient with stage 5b disease on a calcineurin inhibitor and azathioprine you chose NOT to make any changes in immunosuppression.

Consider the following statement:
I would agree with a change in immunosuppression if (choose all that apply):

- ☐ The patient is amenable to modification of immunosuppression
- ☐ There is no evidence of DSA
- ☐ There is a low level of HLA mismatch
- ☐ There has been enough time since transplant
- ☐ I have no concerns about graft function
- ☐ Patient does not have a history of rejection with this graft
- ☐ If CNI trough level is sufficiently high (top 50% of target range for your center)
- ☐ Other

Please define low HLA mismatch: __________________________________

Please explain what other factors your consider: __________________________________

For a patient with stage 5b disease on a calcineurin inhibitor, MMF/MPA, and prednisone you chose NOT to make any changes in immunosuppression.

Consider the following statement:
I would agree with a change in immunosuppression if (choose all that apply):

- ☐ The patient is amenable to modification of immunosuppression
- ☐ There is no evidence of DSA
- ☐ There is a low level of HLA mismatch
- ☐ There has been enough time since transplant
- ☐ I have no concerns about graft function
- ☐ Patient does not have a history of rejection with this graft
- ☐ If CNI trough level is sufficiently high (top 50% of target range for your center)
- ☐ Other

Please define low HLA mismatch: __________________________________

Please explain what other factors your consider: __________________________________

For a patient with stage 5b disease on a calcineurin inhibitor and MMF/MPA you chose NOT to make any changes in immunosuppression.

Consider the following statement:
I would agree with a change in immunosuppression if (choose all that apply):

- ☐ The patient is amenable to modification of immunosuppression
- ☐ There is no evidence of DSA
- ☐ There is a low level of HLA mismatch
- ☐ There has been enough time since transplant
- ☐ I have no concerns about graft function
- ☐ Patient does not have a history of rejection with this graft
- ☐ If CNI trough level is sufficiently high (top 50% of target range for your center)
- ☐ Other

Please define low HLA mismatch: __________________________________

Please explain what other factors your consider:

____

You are taking care of a renal transplant patient with stage 6 disease (one or more high risk cSCC with ~20% risk of nodal metastasis) on the immunosuppression regimen listed on each row. Please mark the next best step regarding change in immunosuppression.

	Stop/Decrease Antimetabolite	Decrease Antimetabolite and Start/Increase Prednisone	Decrease Antimetabolite and Increase Calcineurin Inhibitor	Switch to different antimetabolite	Stop Calcineurin Inhibitor and Start mTOR Inhibitor	Stop Antimetabolite and Start mTOR Inhibitor	No change
Calcineurin Inhibitor + Azathioprine + Prednisone	○	○	○	○	○	○	○
Calcineurin Inhibitor + Azathioprine	○	○	○	○	○	○	○
Calcineurin Inhibitor + MMF/MPA + Prednisone	○	○	○	○	○	○	○
Calcineurin Inhibitor + MMF/MPA	○	○	○	○	○	○	○

For a patient with stage 6 disease on a calcineurin inhibitor, azathioprine, and prednisone you chose NOT to make any changes in immunosuppression.

Consider the following statement:
I would agree with a change in immunosuppression if (choose all that apply):

- ☐ The patient is amenable to modification of immunosuppression
- ☐ There is no evidence of DSA
- ☐ There is a low level of HLA mismatch
- ☐ There has been enough time since transplant
- ☐ I have no concerns about graft function
- ☐ Patient does not have a history of rejection with this graft
- ☐ If CNI trough level is sufficiently high (top 50% of target range for your center)
- ☐ Other

Please define low HLA mismatch:

____

Please explain what other factors your consider:

____

For a patient with stage 6 disease on a calcineurin inhibitor and azathioprine you chose NOT to make any changes in immunosuppression.

Consider the following statement:
I would agree with a change in immunosuppression if (choose all that apply):

- ☐ The patient is amenable to modification of immunosuppression
- ☐ There is no evidence of DSA
- ☐ There is a low level of HLA mismatch
- ☐ There has been enough time since transplant
- ☐ I have no concerns about graft function
- ☐ Patient does not have a history of rejection with this graft
- ☐ If CNI trough level is sufficiently high (top 50% of target range for your center)
- ☐ Other

Please define low HLA mismatch:

____

Please explain what other factors your consider:

____

For a patient with stage 6 disease on a calcineurin inhibitor, MMF/MPA, and prednisone you chose NOT to make any changes in immunosuppression.

Consider the following statement:
I would agree with a change in immunosuppression if (choose all that apply):

- ☐ The patient is amenable to modification of immunosuppression
- ☐ There is no evidence of DSA
- ☐ There is a low level of HLA mismatch
- ☐ There has been enough time since transplant
- ☐ I have no concerns about graft function
- ☐ Patient does not have a history of rejection with this graft
- ☐ If CNI trough level is sufficiently high (top 50% of target range for your center)
- ☐ Other

Please define low HLA mismatch:

____

Please explain what other factors your consider:

____

For a patient with stage 6 disease on a calcineurin inhibitor and MMF/MPA you chose NOT to make any changes in immunosuppression.

Consider the following statement:
I would agree with a change in immunosuppression if (choose all that apply):

- ☐ The patient is amenable to modification of immunosuppression
- ☐ There is no evidence of DSA
- ☐ There is a low level of HLA mismatch
- ☐ There has been enough time since transplant
- ☐ I have no concerns about graft function
- ☐ Patient does not have a history of rejection with this graft
- ☐ If CNI trough level is sufficiently high (top 50% of target range for your center)
- ☐ Other

Please define low HLA mismatch:

____

Please explain what other factors your consider:

____

Please provide any additional comments regarding the scenarios proposed in this survey.

____

Delphi Round 3

Please complete the survey below.

Thank you!

Thank you for your continued participation in this study. A review of consensus achieved in Round 1 and Round 2 of the Delphi consensus panel has been sent to you via email and is summarized in the figure below. Round 3 aims to achieve consensus on patient factors that influence the decision to adjust immunosuppression. This survey can be taken on a mobile device, but will appear clearer and may take less time on a computer.

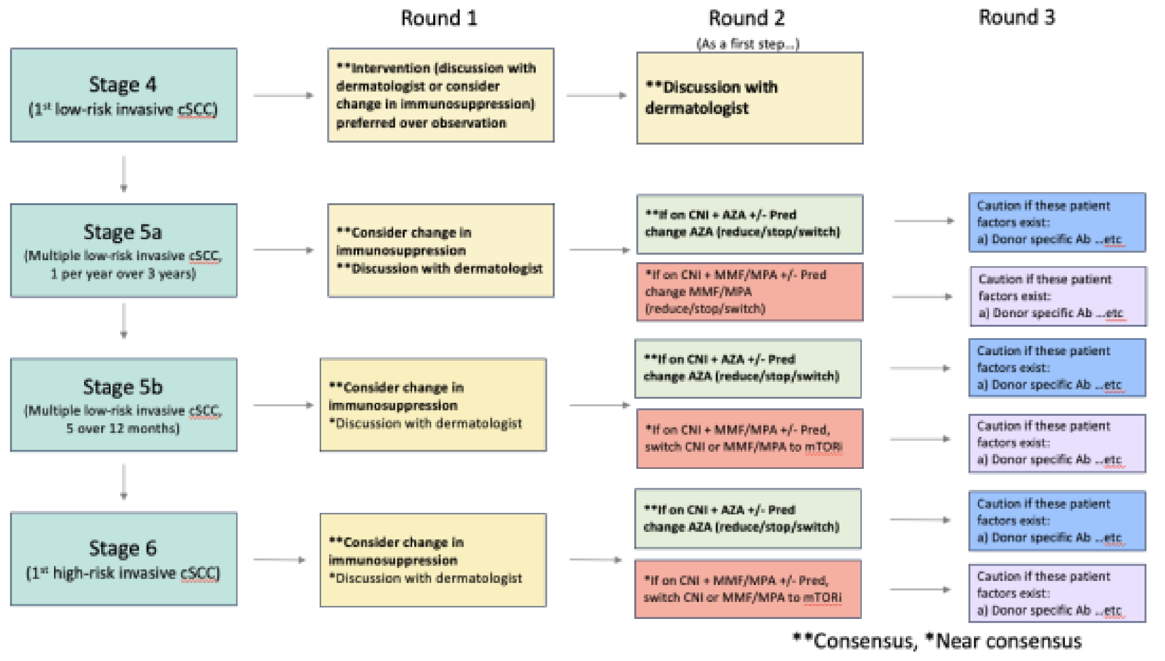

The following questions address medical management for cutaneous squamous cell carcinomas (cSCC) in renal transplant recipients (RTRs). Please select options you consider to be the most acceptable in medical management. For the following questions, please consider a typical RTR in your clinic and assume the specific tumor has already been treated surgically with negative margins.

Definition of Actinic Damage - Skin Cancer Index (AD-SCI) Stages:

Stage 4 First invasive low-risk cSCC (AJCC8 T1-T2; BWH T1-T2a)
Stage 5 Multiple invasive low-risk cSCC (AJCC8 T1-T2; BWH T1-T2a)
5a Slow rate @ approximately 1 per 12 months over last 36 months
5b Fast rate @ approximately 5 over last 12 months
Stage 6 High-risk cSCC (AJCC8 T3; BWH T2b) ~20% risk of nodal metastasis
Massey PR et al. Consensus-Based Recommendations on the Prevention of Squamous Cell Carcinoma in Solid Organ Transplant Recipients: A Delphi Consensus Statement. JAMA Dermatol. 2021 Oct 1;157(10):1219-1226. doi: 10.1001/jamadermatol.2021.3180. PMID: 34468690; PMCID: PMC9937447.

For a renal transplant recipient with stage 5a disease (low-risk, slow-rate cSCC) on CNI + AZA +/- Pred, you consider changing AZA (reduce/stop/switch).

Which of the following features, if present in the patient, would STOP you from making this change? (select all that apply):

- ☐ History of donor specific antibodies (currently undetectable)
- ☐ Current presence of donor specific antibodies
- ☐ Highly sensitized recipient (calculated reaction frequency >80%)
- ☐ Presence of significant proteinuria (urine protein-Cr ratio >50mg/mmol [500mg/g])
- ☐ Marked HLA mismatch
- ☐ Biopsy proven acute T cell rejection within the last 6 months
- ☐ Biopsy proven antibody-mediated rejection within the last 6 months
- ☐ Received transplant within the last 12 months
- ☐ Currently pregnant or potential for pregnancy in the future
- ☐ Other

Please provide your definition of marked HLA mismatch

____

Please describe the other patient factors you would consider:

____

For a renal transplant recipient with stage 5a disease (low-risk, slow-rate cSCC) on CNI + MMF/MPA +/- Pred, you consider changing MMF/MPA (reduce/stop/switch).

Which of the following features, if present in the patient, would STOP you from making this change? (select all that apply):

- ☐ History of donor specific antibodies (currently undetectable)
- ☐ Current presence of donor specific antibodies
- ☐ Highly sensitized recipient (calculated reaction frequency >80%)
- ☐ Presence of significant proteinuria (urine protein-Cr ratio >50mg/mmol [500mg/g])
- ☐ Marked HLA mismatch
- ☐ Biopsy proven acute T cell rejection within the last 6 months
- ☐ Biopsy proven antibody-mediated rejection within the last 6 months
- ☐ Received transplant within the last 12 months
- ☐ Currently pregnant or potential for pregnancy in the future
- ☐ Other

Please provide your definition of marked HLA mismatch

____

Please describe the other patient factors you would consider:

____

For a renal transplant recipient with stage 5b disease (low-risk, fast-rate cSCC) on CNI + AZA +/- Pred, you consider changing AZA (reduce/stop/switch).

Which of the following features, if present in the patient, would STOP you from making this change? (select all that apply):

- ☐ History of donor specific antibodies (currently undetectable)
- ☐ Current presence of donor specific antibodies
- ☐ Highly sensitized recipient (calculated reaction frequency >80%)
- ☐ Presence of significant proteinuria (urine protein-Cr ratio >50mg/mmol [500mg/g])
- ☐ Marked HLA mismatch
- ☐ Biopsy proven acute T cell rejection within the last 6 months
- ☐ Biopsy proven antibody-mediated rejection within the last 6 months
- ☐ Received transplant within the last 12 months
- ☐ Currently pregnant or potential for pregnancy in the future
- ☐ Other

Please provide your definition of marked HLA mismatch

____

Please describe the other patient factors you would consider:

____

For a renal transplant recipient with stage 5b disease (low-risk, fast-rate cSCC) on CNI + MMF/MPA +/- Pred, you consider switching the CNI or MMF/MPA to mTOR inhibitor.

Which of the following features, if present in the patient, would STOP you from making this change? (select all that apply):

- ☐ History of donor specific antibodies (currently undetectable)
- ☐ Current presence of donor specific antibodies
- ☐ Highly sensitized recipient (calculated reaction frequency >80%)
- ☐ Presence of significant proteinuria (urine protein-Cr ratio >50mg/mmol [500mg/g])
- ☐ Marked HLA mismatch
- ☐ Biopsy proven acute T cell rejection within the last 6 months
- ☐ Biopsy proven antibody-mediated rejection within the last 6 months
- ☐ Received transplant within the last 12 months
- ☐ Currently pregnant or potential for pregnancy in the future
- ☐ Other

Please provide your definition of marked HLA mismatch

____

Please describe the other patient factors you would consider:

____

For a renal transplant recipient with stage 6 disease (high-risk cSCC) on CNI + AZA +/- Pred, you consider changing AZA (reduce/stop/switch).<br><br>Which of the following features, if present in the patient, would STOP you from making this change? (select all that apply):	☐ History of donor specific antibodies (currently undetectable)<br>☐ Current presence of donor specific antibodies<br>☐ Highly sensitized recipient (calculated reaction frequency >80%)<br>☐ Presence of significant proteinuria (urine protein-Cr ratio >50mg/mmol [500mg/g])<br>☐ Marked HLA mismatch<br>☐ Biopsy proven acute T cell rejection within the last 6 months<br>☐ Biopsy proven antibody-mediated rejection within the last 6 months<br>☐ Received transplant within the last 12 months<br>☐ Currently pregnant or potential for pregnancy in the future<br>☐ Other
Please provide your definition of marked HLA mismatch	__________________________________
Please describe the other patient factors you would consider:	__________________________________
For a renal transplant recipient with stage 6 disease (low-risk, fast-rate cSCC) on CNI + MMF/MPA +/- Pred, you consider switching the CNI or MMF/MPA to mTOR inhibitor.<br><br>Which of the following features, if present in the patient, would STOP you from making this change? (select all that apply):	☐ History of donor specific antibodies (currently undetectable)<br>☐ Current presence of donor specific antibodies<br>☐ Highly sensitized recipient (calculated reaction frequency >80%)<br>☐ Presence of significant proteinuria (urine protein-Cr ratio >50mg/mmol [500mg/g])<br>☐ Marked HLA mismatch<br>☐ Biopsy proven acute T cell rejection within the last 6 months<br>☐ Biopsy proven antibody-mediated rejection within the last 6 months<br>☐ Received transplant within the last 12 months<br>☐ Currently pregnant or potential for pregnancy in the future<br>☐ Other
Please provide your definition of marked HLA mismatch	__________________________________
Please describe the other patient factors you would consider:	__________________________________
For a renal transplant recipient at any stage, which of the following factors impact your decision to modify immunosuppression?	☐ History of significant sun exposure<br>☐ Overall of burden of pre-malignant skin lesions such as actinic keratoses<br>☐ Previous non-squamous cell carcinoma skin cancer<br>☐ History of non-skin malignancy<br>☐ History of opportunistic infection<br>☐ Older patient age<br>☐ Other

What other factors do you consider?

____

Consider the following statement:

For a renal transplant recipient at any stage, it is recommended to discuss patient management with a dermatologist to optimize skin cancer prevention.

- ◯ Agree
- ◯ Disagree

Please explain your recommendation (optional):

____

ACCORD
Accurate Consensus Reporting Document

Item No.	Section	Checklist Item (*help text*)	Page No.
T1	**Title**	Identify the article as reporting a consensus exercise and state the consensus methods used in the title.<br>*For example, Delphi or nominal group technique.*	Title Page
I1	**Introduction**	Explain why a consensus exercise was chosen over other approaches.	Intro
I2		State the aim of the consensus exercise, including its intended audience and geographical scope (national, regional, global).	Intro
I3		If the consensus exercise is an update of an existing document, state why an update is needed, and provide the citation for the original document.	Intro
M1	**Methods**<br>Registration	If the study or study protocol was prospectively registered, state the registration platform and provide a link. If the exercise was not registered, this should be stated.<br>*Recommended to include the date of registration.*	Not registered
M2	Selection of SC and/or panellists	Describe the role(s) and areas of expertise or experience of those directing the consensus exercise.<br>*For example, whether the project was led by a chair, co-chairs or a steering committee, and, if so, how they were chosen. List their names if appropriate, and whether there were any subgroups for individual steps in the process.*	Methods
M3		Explain the criteria for panellist inclusion and the rationale for panellist numbers. State who was responsible for panellist selection.	Methods
M4		Describe the recruitment process (how panellists were invited to participate).<br>*Include communication/advertisement method(s) and locations, numbers of invitations sent, and whether there was centralised oversight of invitations or if panellists were asked/allowed to suggest other members of the panel.*	Methods
M5		Describe the role of any members of the public, patients or carers in the different steps of the study.	N/A
M6	Preparatory research	Describe how information was obtained prior to generating items or other materials used during the consensus exercise.<br>*This might include a literature review, interviews, surveys, or another process.*	Methods
M7		Describe any systematic literature search in detail, including the search strategy and dates of search or the citation if published already.<br>*Provide the details suggested by the reporting guideline PRISMA and the related PRISMA-Search extension.*	N/A
M8		Describe how any existing scientific evidence was summarised and if this evidence was provided to the panellists.	N/A
M9	Assessing consensus	Describe the methods used and steps taken to gather panellist input and reach consensus (for example, Delphi, RAND-UCLA, nominal group technique).<br>*If modifications were made to the method in its original form, provide a detailed explanation of how the method was adjusted and why this was necessary for the purpose of your consensus-based study.*	Methods
M10		Describe how each question or statement was presented and the response options. State whether panellists were able to or required to explain their responses, and whether they could propose new items.<br>*Where possible, present the questionnaire or list of statements as supplementary material.*	Methods
M11		State the objective of each consensus step.<br>*A step could be a consensus meeting, a discussion or interview session, or a Delphi round.*	Methods
M12		State the definition of consensus (for example, number, percentage, or categorical rating, such as ‘agree’ or ‘strongly agree’) and explain the rationale for that definition.	Methods
M13		State whether items that met the prespecified definition of consensus were included in any subsequent voting rounds.	Methods
M14		For each step, describe how responses were collected, and whether responses were collected in a group setting or individually.	Methods

M15		Describe how responses were processed and/or synthesised.<br>*Include qualitative analyses of free-text responses (for example, thematic, content or cluster analysis) and/or quantitative analytical methods, if used.*	Methods
M16		Describe any piloting of the study materials and/or survey instruments.<br>*Include how many individuals piloted the study materials, the rationale for the selection of those individuals, any changes made as a result and whether their responses were used in the calculation of the final consensus. If no pilot was conducted, this should be stated.*	.Methods
M17		If applicable, describe how feedback was provided to panellists at the end of each consensus step or meeting.<br>*State whether feedback was quantitative (for example, approval rates per topic/item) and/or qualitative (for example, comments, or lists of approved items), and whether it was anonymised.*	Methods
M18		State whether anonymity was planned in the study design. Explain where and to whom it was applied and what methods were used to guarantee anonymity.	Methods
M19		State if the steering committee was involved in the decisions made by the consensus panel.<br>*For example, whether the steering committee or those managing consensus also had voting rights.*	Methods
M20	Participation	Describe any incentives used to encourage responses or participation in the consensus process.<br>*For example, were invitations to participate reiterated, or were participants reimbursed for their time.*	Methods
M21		Describe any adaptations to make the surveys/meetings more accessible.<br>*For example, the languages in which the surveys/meetings were conducted and whether translations or plain language summaries were available.*	N/A
R1	Results	State when the consensus exercise was conducted. List the date of initiation and the time taken to complete each consensus step, analysis, and any extensions or delays in the analysis.	Results
R2		Explain any deviations from the study protocol, and why these were necessary.<br>*For example, addition of panel members during the exercise, number of consensus steps, stopping criteria; report the step(s) in which this occurred.*	N/A
R3		For each step, report quantitative (number of panellists, response rate) and qualitative (relevant socio-demographics) data to describe the participating panellists.	Table 2
R4		Report the final outcome of the consensus process as qualitative (for example, aggregated themes from comments) and/or quantitative (for example, summary statistics, score means, medians and/or ranges) data.	Results, Figures
R5		List any items or topics that were modified or removed during the consensus process. Include why and when in the process they were modified or removed.	N/A
D1	Discussion	Discuss the methodological strengths and limitations of the consensus exercise.<br>*Include factors that may have impacted the decisions (for example, response rates, representativeness of the panel, potential for feedback during consensus to bias responses, potential impact of any non-anonymised interactions).*	Discussion
D2		Discuss whether the recommendations are consistent with any pre-existing literature and, if not, propose reasons why this process may have arrived at alternative conclusions.	Discussion
O1	Other information	List any endorsing organisations involved and their role.	***
O2		State any potential conflicts of interests, including among those directing the consensus study and panellists. Describe how conflicts of interest were managed.	***
O3		State any funding received and the role of the funder.<br>*Specify, for example, any funder involvement in the study concept/design, participation in the steering committee, conducting the consensus process, funding of any medical writing support. This could be disclosed in the methods or in the relevant transparency section of the manuscript. Where a funder did not play a role in the process or influence the decisions reached, this should be specified.*	Title page

ACCORD
Accurate Consensus Reporting Document

From: PLoS Med 21(1): e1004326. https://doi.org/10.1371/journal.pmed.1004326 For more information see: https://www.ismpp.org/accord
